# A native parasitic plant and soil microorganisms facilitate a native plant co‐occurrence with an invasive plant

**DOI:** 10.1002/ece3.5407

**Published:** 2019-07-04

**Authors:** Junmin Li, Ayub M. O. Oduor, Feihai Yu, Ming Dong

**Affiliations:** ^1^ Zhejiang Provincial Key Laboratory of Plant Evolutionary Ecology and Conservation Taizhou University Taizhou China; ^2^ Key Laboratory of Hangzhou City for Ecosystem Protection and Restoration, College of Life and Environmental Sciences Hangzhou Normal University Hangzhou China; ^3^ Department of Applied and Technical Biology Technical University of Kenya Nairobi Kenya

**Keywords:** biotic resistance, coexistence, invasive plants, native plants, parasitic plants, soil microbes

## Abstract

Invasive plants often interact with antagonists that include native parasitic plants and pathogenic soil microbes, which may reduce fitness of the invaders. However, to date, most of the studies on the ecological consequences of antagonistic interactions between invasive plants and the resident biota focused only on pairwise interactions. A full understanding of invasion dynamics requires studies that test the effects of multiple antagonists on fitness of invasive plants and co‐occurring native plants. Here, we used an invasive plant *Mikania micrantha*, a co‐occurring native plant *Coix lacryma‐jobi,* and a native holoparasitic plant *Cuscuta campestris* to test whether parasitism on *M. micrantha* interacts with soil fungi and bacteria to reduce fitness of the invader and promote growth of the co‐occurring native plant. In a factorial setup, *M. micrantha* and *C. lacryma‐jobi* were grown together in pots in the presence versus absence of parasitism on *M. micrantha* by *C. campestris* and in the presence versus absence of full complements of soil bacteria and fungi. Fungicide and bactericide were used to suppress soil fungi and bacteria, respectively. Findings show that heavy parasitism by *C. campestris* caused the greatest reduction in *M. micrantha* biomass when soil fungi and bacteria were suppressed. In contrast, the co‐occurring native plant *C. lacryma‐jobi* experienced the greatest increase in biomass when grown with heavily parasitized *M. micrantha* and in the presence of a full complement of soil fungi and bacteria. Taken together, our results suggest that selective parasitism on susceptible invasive plants by native parasitic plants and soil microorganisms may diminish competitive ability of invasive plants and facilitate native plant coexistence with invasive plants.

## 1 INTRODUCTION

Invasion of native communities by exotic plant species is a major element of global environmental change reducing native plant diversity (Kourtev, Ehrenfeld, & Häggblom, 2003; Mack et al., 2000; Vila et al., 2011). Within their introduced ranges, invasive plants often interact with a new suite of antagonists such as native parasitic plants (Li, Jin, & Song, 2012; Miao et al., 2012; Prider, Walting, & Facelli, 2009; Wang, Guan, Li, Yang, & Li, 2012; Yu, Liu, He, Miao, & Dong, 2011; Yu, Yu, Miao, & Dong, 2008) and soil‐borne pathogens (Mitchell et al., 2006). The invasive plants may also interact with soil‐borne microbial mutualists (Kowalski et al., 2015; Richardson, Allsopp, D'antonio, Milton, & Rejmánek, 2000; Simberloff & Von Holle, 1999). The antagonists and mutualists may individually and interactively influence fitness of invasive plants (Hill & Kotanen, 2012; Mitchell et al., 2006). Although the ecological consequences of antagonistic interactions between invasive plants and the resident biota are well documented (Hill & Kotanen, 2012; Levine, Adler, & Yelenik, 2004; Maron & Vilà, 2001; Vila et al., 2011), most of such studies focused only on single interaction types, when in reality, multiple interactions occur simultaneously (van Kleunen, Bossdorf, & Dawson, 2018). A full understanding of invasion dynamics requires studies that test the effects of multiple antagonists on fitness of invasive plants and co‐occurring native plants (van Kleunen et al., 2018; Oduor, 2013; Oduor, Kleunen, & Stift, 2017).

Soil microbial communities may influence individual plant fitness, plant community succession, and invasion by acting as plant pathogens and mutualists (Moora & Zobel, 1996; van der Putten, Klironomos, & Wardle, 2007; Shivega & Aldrich‐Wolfe, 2017). Mycorrhizal fungi and nitrogen‐fixing microbes are the two main groups of plant mutualists (van Kleunen et al., 2018). They can benefit plants by facilitating the availability of major plant nutrients and producing plant growth‐promoting substances (Batten, Scow, Davies, & Harrison, 2006). On the other hand, pathogenic microbes reduce plant fitness (Callaway, Thelen, Rodriguez, & Holben, 2004; Chen et al., 2018; Klironomos, 2002; Maron, Marler, Klironomos, & Cleveland, 2011; van der Putten, Dijk, & Peters, 1993). There is mixed empirical evidence on associations between invasive plants and microbial mutualists. Studies in grasslands and mixed‐grass prairie of North America found that invasive and naturalized alien plants had fewer and weaker associations with arbuscular mycorrhizal (AM) fungi than native plant species (Jordan, Aldrich‐Wolfe, Huerd, Larson, & Muehlbauer, 2012; Pringle et al., 2009; Sigüenza, Crowley, & Allen, 2006; Vogelsang & Bever, 2009). These and other findings that did not find dependency of invasive plants on mycorrhizal fungi led to a suggestion that reduced dependency on microbial mutualists may be an important feature of invasiveness of exotic plants (the degraded mutualism hypothesis; Bunn, Ramsey, & Lekberg, 2015). In contrast, studies in other ecosystems in Europe, New Zealand, and South America (e.g., Dickie, Bolstridge, Cooper, & Peltzer, 2010; Menzel et al., 2017; Nuñez & Dickie, 2014; Štajerová, Šmilauerová, & Šmilauer, 2009) found a majority of exotic plant species to be mycorrhizal. The conflicting results suggest that whether exotic plants benefit from being mycorrhizal may depend upon the plant taxa and ecological context. Associations between invasive plants with nitrogen‐fixing bacteria have also been reported (Le Roux, Hui, Keet, & Ellis, 2017). Invasive plants have also been shown to suffer less from negative effects of pathogenic soil biota than co‐occurring native plant species (Agrawal et al., 2005; Kardol, Cornips, Kempen, Bakx‐Schotman, & Putten, 2007; Klironomos, 2002; Kulmatiski, Beard, Stevens, & Cobbold, 2008). Nevertheless, more recent studies suggest that exotic plants can accumulate soil pathogens over time, which could potentially reduce their impacts on native plants (Diez et al., 2010; Dostál, Müllerová, Pyšek, Pergl, & Klinerová, 2013; Speek et al., 2015; Stricker, Harmon, Goss, Clay, & Luke Flory, 2016). Thus, the net impact of soil microbes (negative, neutral or positive) on fitness of invasive plants and co‐occurring native plants may depend upon the balance of positive effects of mutualists and negative effects of pathogens present in a particular soil (Klironomos, 2002; van der Putten et al., 2013; Westover & Bever, 2001).

As parasitic plants are common in natural communities (Pennings & Callaway, 2002), invasive plants may interact simultaneously with native plants, soil microbes, and native parasitic plants (Li, Jin, Hagedorn, & Li, 2014). Empirical studies have shown that soil microbial communities can mediate competitive interactions between invasive plants and native plants (e.g., Allen, Meyerson, Flick, & Cronin, 2018; Lankau, 2010; Marler, Zabinski, & Callaway, 1999; Shivega & Aldrich‐Wolfe, 2017). For example, rhizospheric soil biota of the invader *Phragmites australis* increased biomass of a native plant *Spartina alterniflora* when the two plant species were grown in competition with each other (Allen et al., 2018). In a separate study, microbial taxa inhibited the allelopathic effect of the invader *Alliaria petiolata* on seedlings of the native plant *Platanus occidentalis* (Lankau, 2010). In pairwise competition experiments that compared performance of two native prairie plants (*Oligoneuron rigidum* and *Andropogon gerardii*) against one invader (*Carduus acanthoides*), the native plants fared better against the invader in the presence of a native microbial community (Shivega & Aldrich‐Wolfe, 2017). AM fungi increased the negative effects of the invader *Centaurea maculosa* on a native bunchgrass *Festuca idahoensis* (Marler et al., 1999). Studies have also shown that native parasitic plants can affect competition between invasive host plants and co‐occurring native plants. For instance, native holoparasitic plants such as *Cuscuta campestris* (Yu et al., 2008), *C. australis* (Li et al., 2012; Wang et al., 2012; Yu et al., 2011), and *Cassytha pubescens* (Prider et al., 2009) caused more damage to their invasive host species than co‐occurring native species. Thus, the holoparasitic plants have been suggested as a potential biological control agent against the plant invaders (Miao et al., 2012). However, previous work only examined the separate effects of soil microbes and native parasitic plants on interactions between invasive plants and native plants. Therefore, whether soil microbial community and native parasitic plants operate independently or interact in ways that exacerbate or ameliorate the effects of each other to influence competitive interactions between invasive plants and native plants remains unexplored.

Here, we used an invasive plant *Mikania micrantha*, a co‐occurring native plant *Coix lacryma‐jobi*, and a native holoparasitic plant *C. campestris* to address the question: Can parasitism on an invasive plant by a native holoparasitic plant interact with soil fungi and bacteria to reduce fitness of the invader and promote growth of a co‐occurring native plant?

## 2 MATERIALS AND METHODS

### 2.1 Study plant species

*Mikania micrantha* (Asteraceae) (hereinafter *Mikania*) is native to Central and South America and was introduced into China in 1919 (Holm, Plucknett, Pancho, & Herberger, 1977). At present, *Mikania* is distributed widely in Guangdong province in South China where it is invasive (Zhang, Ye, Cao, & Feng, 2004). *Cuscuta campestris* (hereinafter *Cuscuta*) is native to China and occurs in the provinces of Fujian, Guangdong, and Xinjiang Uygur Autonomous Region, China (Wang, Wang, & Liao, 2004). As a holoparasitic plant, *Cuscuta* acquires some or all of its water, carbon, and nutrients via the vascular tissue of the hosts' roots or shoots, which significantly inhibits growth of the host. Previous field observations and greenhouse experiments showed that *Cuscuta* preferentially parasitized *Mikania* relative to native plants, which significantly reduced growth and cover of the invader and facilitated native species diversity in invaded patches (Shen, Hong, Ye, Cao, & Wang, 2007; Wang et al., 2004; Yu et al., 2008). The native plant *Coix lacryma‐jobi* (Poaceae) (hereinafter *Coix*) was chosen for this experiment because it was the most common native species that co‐occurred with *Mikania* in the invaded community. Results of a previous field survey suggest that parasitism by *Cuscuta* may reduce competitive exclusion of *Coix* by *Mikania* (Li et al., 2014).

### 2.2 Location of study

A common garden pot experiment was conducted in Dengshuiling village, in the southeast of Dongguan City (113°31′‐114°15′E; 22°39′‐23°09′N), Guangdong Province, China. The province has a subtropical climate with a mean annual precipitation of 1,819.9 mm, temperature of 23.1°C, and sunshine time of 1,873.7 hr. *Mikania* first invaded the province in early 1990s where it spread extensively in the shrublands and abandoned agricultural fields.

### 2.3 Preparation of experimental plant and soil materials

We collected soil from a field near Dengshuiling village. Ten 1 m × 1 m plots were chosen randomly in an abandoned agricultural field site without *Mikania*. Vegetation and litter were removed from the upper soil surface, and then, soil (red clay) was collected at depths of 0–15 cm from the plots. The soil was mixed with sand (3:1, soil/sand) and homogenized before use. This mixture enabled us to maintain good drainage and accurately harvest roots at the end of the experiment.

We obtained stem cuttings of *Mikania* from multiple maternal families in a field near Dengshuiling village on 16 July 2006 and then propagated them for use in the experimental setup described below. Sharp pruning shears (sterilized with 70% ethanol) were used to generate the cuttings from upper intact plant parts. Each cutting measured 10 cm in length, and its leaf count was reduced by a half to reduce water loss upon transplant. The cuttings were then inserted into a potted soil (up to a third of the entire length), with the stem maintained in a vertical orientation. *Coix* was raised from seeds that had been purchased from Shandong Heze Chinese Medicine Institute in March 2006. In order to eliminate any pathogen that might have been present on the *Coix* seeds, the seeds were surface‐sterilized as follows. The seeds were immersed in 20% CuSO_4_ for 10 min and later soaked in water for 24 hr, 70% ethanol for 1 min, water again for 5 min, 10% H_2_O_2_ for 5 min, and finally rinsed with sterilized water three times (see Li et al., 2014). In June 2006, we sowed similar‐sized seeds in plastic‐plug trays filled with soil of the same source as above. The soil was sterilized before use to prevent any microbes present in the soil from influencing early growth of *Coix* seedlings and *Mikania* cuttings.

### 2.4 Experimental setup

To test whether parasitism by *Cuscuta* on *Mikania* interacted with soil fungi and bacteria to influence competitive interactions between *Mikania* and *Coix*, we performed a factorial pot experiment. In the experiment, we grew an individual *Coix* in competition with *Mikania* (parasitized vs. not parasitized), and when soil fungi and bacteria were suppressed versus not suppressed. In late July 2006, individual *Mikania* cuttings and *Coix* seedlings (each measured *c*. 15 cm in length) that had been raised as described above were carefully removed from the nursery without destroying the roots and transplanted into 3‐L pots (25 cm in diameter) that had been filled with nonsterilized soil from the same source as above. Within the pot, *Mikania* and *Coix* were spaced 15 cm apart. Immediately after transplant, the pots were placed under a shade tree to avoid excess evapotranspiration. Then, three days later, the pots were moved to an open‐ field common garden. A week after transplant, bamboo sticks (1 m long) were driven into the soil near *Mikania* to provide support because *Mikania* is a climber species. The plants were fertilized with 50% strength Hoagland's nutrient solution once a week. Throughout the experiment, the plants were watered twice a day with tap water.

Three weeks after transplant, *Cuscuta* stems were collected from a field near the village of Dengshuiling and wound around *Mikania* stems (Figure S1). We used *Cuscuta* raised from stem cuttings instead of seeds because there were no mature seeds in the field at the start of the experiment. To represent low‐ and high‐level parasitism, we wound one and three *Cuscuta* stems (each 15 cm long), respectively, around *Mikania* stems. As a control, we grew *Mikania* without *Cuscuta* infestation. We did not infest *Coix* with *Cuscuta* because in the habitat where we sampled experimental plant materials, *Cuscuta* avoided *Coix* (although *Coix* experienced *c*. 2.5% of parasitism relative to *Mikania* in other habitats). To suppress fungi that were present in the potted soil, we applied benomyl (purchased from Yida Chemical Inc.). Benomyl had been shown to effectively reduce soil fungi including AM fungi with negligible direct effects on plants (Callaway, Mahall, Wicks, Pankey, & Zabinski, 2003; Hetrick, Wilson, & Hartnett, 1989). The fungicide was applied at a concentration of 50 mg benomyl/kg soil (Callaway et al., 2003; Hetrick et al., 1989). We used streptomycin sulfate (purchased from Linhai Seeds and Vegetation Company) to suppress bacteria in the potted soil. Streptomycin is a commonly used bactericidal antibiotic (El‐Khair & Haggag, 2007) that acts by interfering with normal protein synthesis in bacteria (Bailey, Smith, & Bolton, 2003). We added 40,000 titer units of streptomycin sulfate/kg soil to the soil in the pot every week. The fungicide and bactericide were solubilized in tap water and applied at the rate of 100 ml per pot. As a control against the fungicide and bactericide treatments, we applied 100 ml of tap water. Each of the resulting 12 treatment combinations (i.e., three levels of parasitism on *Mikania* by *Cuscuta* [no parasitism, light parasitism, and heavy parasitism] × 2 levels of fungicide [applied vs. not applied] × 2 levels of bactericide [applied vs. not applied]) was replicated five times, resulting in 60 experimental pots. The pots were arranged randomly within the garden and the experiment ran for 7 weeks.

### 2.5 Measurements

We terminated the experiment at the end of week seven. We then separated *Cuscuta* from *Mikania* and harvested individual *Mikania* and *Coix* plants separately. We separated roots and shoots of the experimental plants and then dried them to a constant biomass at 80°C for 48 hr. We then determined total biomass (root and shoot) of the dried plant materials.

At harvest, we determined whether fungicide application had suppressed soil fungi by examining root colonization of all the experimental *Mikania* and *Coix* plants by AM fungi. We did this before the plant materials were oven‐dried. From each individual plant, we obtained fine roots that were then cut into 1‐cm‐long segments and fixed using formalin/acetic acid/alcohol (FAA) fixative solution. Root samples were cleaned with 10% KOH solution at 90°C for 40 min, acidified in 2% HCl for 5 min, stained with 0.01% acid fuchsin (Kormanik, Bryan, & Schultz, 1980), and then observed under a microscope for presence of AM fungi. We considered a root segment to have AM fungi when it had arbuscules in the cortical cells. For every individual plant, we then determined percentage colonization by AM fungi as follows: AM fungi colonization (%) = 100 × (infected root length/observed root length).

We also determined whether bactericide application had suppressed soil bacteria in the experimental soil material. To do so, we obtained soil samples from individual experimental pots after the plants had been harvested. The soil samples were then stored at 4°C and transported to the laboratory immediately. The soil was then sieved using a sterilized 2‐mm sieve to remove any debris. The number of colony‐forming units (CFUs) in each soil sample was then directly calculated using acridine orange fluorescent staining method under DMLS Fluorescence microscope (Leica Mikrosysteme Vertrieb GmbH Mikroskopie und Histologie; Li & Jin, 2006). To avoid contamination, all the equipments used for processing soil samples were sterilized and cleaned with 70% ethanol before and between uses.

### 2.6 Statistical analysis

We used a three‐way analysis of variance (ANOVA) to test whether parasitism on *Mikania* by *Cuscuta* (three levels: no parasitism, light parasitism, and heavy parasitism), soil fungi (suppressed vs. not suppressed), and soil bacteria (suppressed vs. not suppressed) had main and interactive effects on biomass yield of *Mikania* and *Coix*. Parasitism, fungicide, and bactericide were specified as independent variables, while total biomass of *Mikania* and *Coix* (root and shoot combined) was specified as a dependent variable. We also used ANOVA to test whether colonization of *Mikania* and *Coix* roots by AM fungi differed significantly between fungicide treatments, and whether the number of soil bacteria differed between bactericide treatments. In the cases where there were significant main and interactive effects of parasitism, soil fungi, and soil bacteria on the growth of *Mikania* and *Coix*, root colonization by AM fungi, and the number of CFUs of soil bacteria, we performed post hoc least‐squares means comparisons between the treatment levels (*α* = 0.05%). All statistical analyses were performed in SPSS v.16.0. All the figures were generated in Sigma Plot v.11.0.

## 3 RESULTS

### 3.1 Biomass of the invasive plant *Mikania*

**Figure 1:**
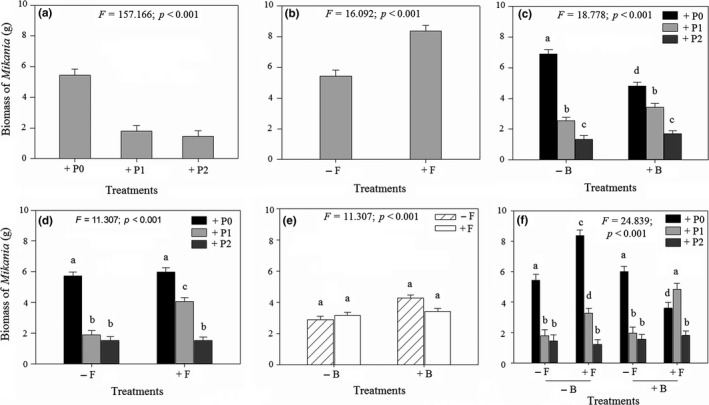
Mean (±1 *SE*) biomass of *Mikania micrantha* plants grown in the presence of *Coix lacryma‐jobi* under different levels of parasitism by *Cuscuta campestris* and in the presence versus absence of a full complement of soil fungi and bacteria. Fungicide and bactericide were used to suppress soil fungi and bacteria, respectively. (a) Main effect of different levels of parasitism: +P0, +P1, and +P2 indicate no parasitism, light parasitism, and heavy parasitism on *C. campestris*, respectively; (b) main effect of fungicide; −F indicates without fungicide, +F indicates with fungicide; (c) interactive effects of different level of parasitism and bactericide; (d) interactive effect of different level of parasitism and fungicide; (e) interactive effect of different level of bactericide and fungicide; (f) interactive effect of different level of parasitism, bactericide, and fungicide. Significance of the main and interactive effects was determined by three‐way ANOVA tests (*cf*. Table S1). Letters above bars indicate the results of post hoc least‐squares mean comparisons (bars that do not share a letter are significantly different)

Parasitism by *Cuscuta* on *Mikania* significantly reduced biomass of the invader (Figure 1a; Table S1). However, heavy and light parasitism caused similar declines in biomass (Figure 1a). Suppression of soil bacteria improved *Mikania* biomass, although not significantly (Table S1). *Mikania* produced more biomass when soil fungi were suppressed than when not suppressed (significant main effect of fungicide Figure 1b; Table S1). Soil fungi and bacteria modified the effects of parasitism on *Mikania* (significant two‐way interactions: parasitism × bactericide; parasitism × fungicide; Figure 1c,d; Table S1). In the presence of a full complement of soil bacteria (bactericide not applied), light and heavy parasitism by *Cuscuta* reduced *Mikania* biomass by 62% and 79%, respectively (Figure 1c). However, when bacteria were suppressed (bactericide applied), light and heavy parasitism by *Cuscuta* reduced *Mikania* biomass by 31% and 66%, respectively (Figure 1c). Similarly, in the presence of a full complement of soil fungi (fungicide not applied), light and heavy parasitism by *Cuscuta* reduced *Mikania* biomass by 68% and 72%, respectively (Figure 1d). On the other hand, when fungi were suppressed (fungicide applied), light and heavy parasitism by *Cuscuta* reduced *Mikania* biomass by 35% and 75%, respectively (Figure 1d). Soil bacteria influenced the effect of soil fungi on *Mikania* biomass (significant interaction between bactericide and fungicide; Figure 1e; Table S1). When bacteria were not suppressed, *Mikania* produced more biomass when fungi were suppressed than when not suppressed (Figure 1e). However, when bacteria were suppressed, the opposite pattern was observed (Figure 1e). Bacteria and fungi jointly influenced the suppressive effects of *Cuscuta* on *Mikania* (significant three‐way interaction: parasitism × bactericide × fungicide; Figure 1f; Table S1). Heavy parasitism by *Cuscuta* caused the greatest decline in *Mikania* biomass (−85.3%) when fungi were suppressed while bacteria were not suppressed (Figure 1f).

### 3.2 Biomass of the native plant *Coix*

**Figure 2:**
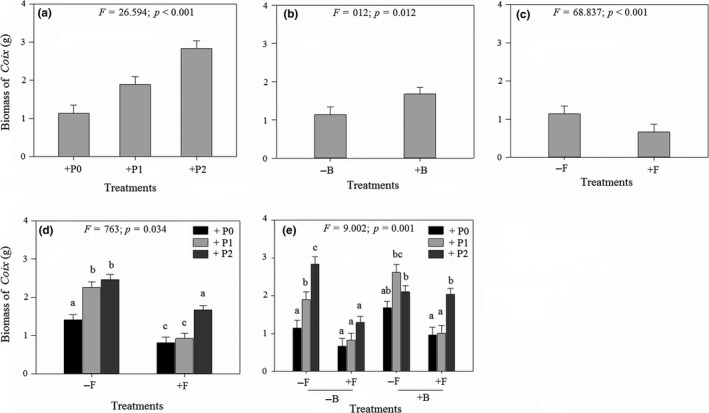
Mean (±1 *SE*) biomass of *Coix lacryma‐jobi* plants grown with *Mikania micrantha* plants that were parasitized by *Cuscuta campestris* at different intensities and in the presence versus absence of a full complement of soil fungi and bacteria. Fungicide and bactericide were used to suppress soil fungi and bacteria, respectively. (a) Main effect of parasitism by *Cuscuta campestris*: +P0, +P1, and +P2 indicate no parasitism, light parasitism, and heavy parasitism on *C. campestris*, respectively; (b) Main effect of bactericide: −B indicates without bactericide, +B indicates with bactericide; (c) main effect of fungicide: −F indicates without fungicide, +F indicates with fungicide; (d) interactive effect of parasitism on *C. campestris* and fungicide; (e) interactive effect of parasitism on *C. campestris*, fungicide, and bactericide. Significance of the main and interactive effects was determined by three‐way ANOVAs tests (*cf*. Table S2). Letters above bars indicate the results of post hoc least‐squares mean comparisons (bars that do not share a letter are significantly different)

Biomass of the native plant *Coix* was significantly higher in treatments where *Mikania* was parasitized (light and heavy) than in the absence of parasitism (Figure 2a and Table S2). Suppression of soil bacteria caused a significant increase in *Coix* biomass (significant main effect of bactericide; Figure 2b and Table S2). However, suppression of fungi caused a significant decline in *Coix* biomass (significant main effect of fungicide; Figure 2c and Table S2). Joint suppression of fungi and parasitism on *Mikania* influenced *Coix* biomass (significant two‐way interaction: parasitism × fungicide; Figure 2d and Table S2). When the full complement of soil fungi was present, *Coix* produced similar biomass under light and heavy levels of parasitism (Figure 2d). However, when fungi were suppressed, *Coix* produced significantly higher biomass when *Mikania* was heavily parasitized than in the absence of parasitism and under light parasitism (Figure 2d). *Coix* biomass was also influenced by the joint effects of parasitism on *Mikania* and soil fungi and bacteria (significant three‐way interaction: parasitism × bactericide × fungicide; Figure 2e and Table S2). *Coix* experienced the greatest gain in biomass (163.6%) when *Mikania* was heavily parasitized and in the presence of a full complement of soil fungi and bacteria (Figure 2e). In contrast, *Coix* experienced a marginal gain in biomass when either fungi or bacteria were suppressed despite heavy parasitism on *Mikania* (Figure 2e).

### 3.3 Effects of fungicide and bactericide on AM fungi and soil bacteria

**Figure 3:**
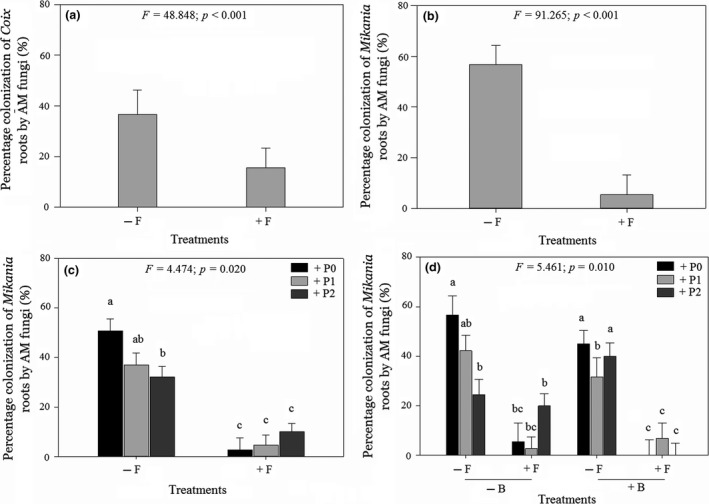
Mean (±1 *SE*) AM mycorrhizal colonization levels of *Coix lacryma‐jobi* and *Mikania mirantha* roots in the presence of different levels of parasitism on *M. micrantha* by *Cuscuta campestris* and in the presence versus absence of a full complement of soil fungi and bacteria. Fungicide and bactericide were used to suppress soil fungi and bacteria, respectively. (a) Main effect of fungicide on the mycorrhizal colonization level of *Coix* root; (b) main effect of fungicide on the mycorrhizal colonization level of *Mikania* root; (c) interactive effects of different levels of parasitism and fungicide on the AM fungal colonization of *Mikania* root; (d) interactive effects of different level of parasitism, bactericide, and fungicide on the AM fungal colonization of *Mikania* root. Significance of the main and interactive effects was determined by three‐way ANOVAs tests (*cf*. Tables S3 and S4). Letters above bars indicate the results of post hoc least‐squares mean comparisons (bars that do not share a letter are significantly different)

**Figure 4:**
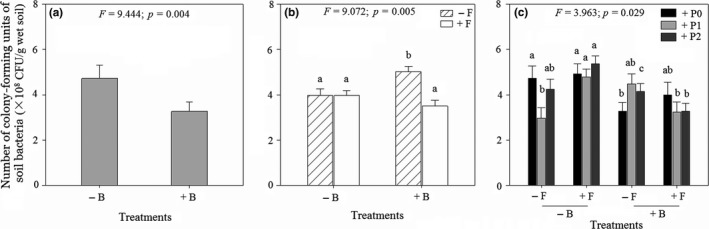
Mean (±1 *SE*) number of colony‐forming units (CFUs) of soil bacteria in a pot with *Coix lacryma‐jobi* and *Mikania mcirantha* in the presence of different levels of parasitism on *M. micrantha* by *Cuscuta campestris* and soil fungi and bacteria. Fungicide and bactericide were used to suppress soil fungi and bacteria, respectively. (a) Main effect of bactericide; (b) interactive effect of bactericide and fungicide; (c) interactive effect of parasitism, bactericide, and fungicide. Significance of the main and interactive effects was determined by three‐way ANOVAs tests (*cf*. Table S5). Letters above bars indicate the results of post hoc least‐squares mean comparisons (bars that do not share a letter are significantly different)

The addition of fungicide significantly reduced colonization of *Coix* and *Mikania* roots by AM fungi (Figure 3a,b; Table S3). Fungicide application modified the effect of *Cuscuta* on colonization of *Mikania* roots by AM fungi (significant two‐way interaction: parasitism × fungicide; Figure 3c; Table S3). When fungicide was not applied, light and heavy parasitism by *Cuscuta* had similar effects on colonization by AM fungi, although both parasitism levels caused significant declines in colonization relative to no parasitism (Figure 3c). However, when fungicide was applied, colonization by AM fungi was similar across parasitism levels (Figure 3c). Application of bactericide modified the joint effects of fungicide and parasitism on colonization of *Mikania* by AM fungi (significant three‐way interaction: parasitism × bactericide × fungicide; Figure 3d and Table S3). *Mikania* experienced the highest level of colonization (58%) in the absence of parasitism and when fungicide and bactericide were not applied (Figure 3d). In contrast, colonization was lowest when both fungicide and bactericide were applied (Figure 3d). For *Coix*, parasitism and bactericide did not influence root colonization by AM fungi (Table S4). Similar to the effects of fungicide on colonization by AM fungi, the addition of bactericide significantly reduced the number of CFUs of soil bacteria (Figure 4a; Table S5). Addition of fungicide modified the effect of bactericide on the number of CFUs (significant two‐way interaction: bactericide × fungicide; Figure 4b; Table S5). When bactericide was not added, the number of CFUs was similar between pots where fungicide was applied and in pots without fungicide (Figure 4b). However, when bactericide was applied, pots without fungicide had significantly higher number of CFUs than pots with fungicide (Figure 4b). Parasitism on *Mikania* by *Cuscuta* influenced the effects of both fungicide and bactericide on the numbers of CFUs (significant three‐way interaction: parasitism × bactericide × fungicide; Figure 4c and Table S5). The mean number of CFUs was highest (5.37 × 10^8^ CFU/g wet soil) when *Mikania* was subjected to heavy parasitism by *Cuscuta* and when fungicide was added but bactericide not added to the pot (Figure 4c). However, the mean number of CFUs was lowest (2.97 × 10^8^ CFU/g wet soil) when neither fungicide nor bactericide was added to the pot and in the presence of light parasitism on *Mikania* by *Cuscuta* (Figure 4c).

## 4 DISCUSSION

The factorial manipulation of soil fungi and bacteria and parasitism on the invasive plant *Mikania* by a native holoparasite *Cuscuta* permitted us to measure the relative strengths and combined effects of parasitism and soil microbial community on interaction between an invasive plant and a co‐occurring native plant. Parasitism on *Mikania* by *Cuscuta* caused a significant decline in biomass of the invader, although the magnitude of impact was modified by the presence of fungi and bacteria in the soil. More specifically, heavy parasitism by *C. campestris* caused the greatest reduction in *M. micrantha* biomass when soil fungi and bacteria were suppressed (Figure 1f). In contrast, the co‐occurring native plant *Coix* experienced the greatest gain in biomass when *Mikania* was heavily parasitized and in the presence of a full complement of soil bacteria and fungi (Figure 2e). *Mikania* had the highest level of root colonization by AM fungi in the absence of parasitism and in the presence of a full complement of soil bacteria and fungi (Figure 3d). In contrast, colonization of *Coix* by AM fungi was not influenced by parasitism on its competitor *Mikania* or by the presence of soil bacteria (Figure 3d). Heavy parasitism on *Mikania* by *Cuscuta* and suppression of soil fungi stimulated bacterial growth in the experimental pots (Figure 4c). Overall, these results suggest that heavy parasitism by *Cuscuta* and soil bacteria had synergistic negative effects on growth of *Mikania*, while the co‐occurring *Coix* benefitted under the same growth conditions. More broadly, the results suggest that native parasitic plants and soil microorganisms can synergistically facilitate coexistence of native plants with invasive plants. Through selective patterns of parasitism by native parasitic plants and in the presence of soil microbes, susceptible invasive hosts may exhibit diminished competitive ability, while co‐occurring nonhost (or less preferred) native species increase in dominance.

### 4.1 The interactions between parasitism on *Mikania* by *Cuscuta*, soil microbes, and the native plant *Coix*

Heavy parasitism by *Cuscuta* had the greatest negative effect on *Mikania* growth when soil fungi were suppressed and in the presence of a full complement of soil bacteria (Figure 1e), which suggests that heavy parasitism weakened defense of *Mikania* against pathogenic bacteria that were likely present in the soil. The results also suggest that suppressing soil fungi eliminated or reduced beneficial effects of fungal mutualists of *Mikania*. Parasitic plants can affect growth of their hosts by extracting resources such as water, nutrients, and organic compounds from the host's vascular system (Press, Scholes, & Watling, 1999). Because these same resources are used by plants to make secondary metabolites that have been shown to be toxic to plant pathogens (Bouwmeester, Roux, Lopez‐Raez, & Becard, 2007), it is likely that heavily parasitized *Mikania* individuals had low concentrations of secondary metabolites and consequently low resistance against pathogenic bacteria that were likely present in the experimental soil. This hypothesis is plausible because species in the genus *Cuscuta* have been shown to be powerful sinks of host photosynthates and nutrients and can therefore preclude host allocation of resources to growth, stress tolerance, or defense (Jeschke, Bäumel, & Räth, 1994; Shen, Xu, Hong, Wang, & Ye, 2013). The apparent synergistic negative effects of *Cuscuta* and soil bacteria on *Mikania* likely released the native plant *Coix* from strong competition from *Mikania* as *Coix* experienced the greatest gain in biomass under similar growth conditions, although when soil fungi were not suppressed (Figure 2e).

*Mikania* had the highest level of root colonization by AM fungi in the absence of parasitism by *Cuscuta* and in the presence of a full complement of soil fungi and bacteria (Figure 3d). This result supports findings on other study systems that infection by parasitic plants can reduce root colonization by AM fungi (Davies & Graves, 1998; Gehring & Whitham, 1992; McKibben & Henning, 2018). The causal mechanism might be a reduced carbon availability (Davies & Graves, 1998). Given that AM fungi and parasitic plants are both carbon sinks (Davies & Graves, 1998), dual infection could lead to the AM fungi and parasitic plants competing for carbon from the host plant. If the parasitic plant is a superior competitor, the reduction in available carbon resources may feedback to disrupt interactions between the host plant and fungal mutualists of the plant (Davies & Graves, 1998; Press & Phoenix, 2005; Stewart & Press, 1990). In support of this, biomass production in *Mikania* plants parasitized by *Cuscuta* was significantly reduced relative to nonparasitized *Mikania* (Figure 1f), suggesting that *Cuscuta* suppressed the AM fungi through a reduction in the available carbon. Future mechanistic experiments should directly test whether parasitism on *Mikania* by *Cuscuta* reduces carbon allocation to AM fungi.

Colonization of *Mikania* roots by AM fungi was lowest in the presence of parasitism by *Cuscuta* and when soil fungi and bacteria were suppressed (Figure 3d). In contrast, for the native plant *Coix* that grew with *Mikania* in the same pot, only fungicide application reduced root colonization by AM fungi (Figure 3a). These contrasting results could be explained both by the absence of parasitism on *Coix* by *Cuscuta* and suppressive effects of the fungicide and bactericide. As *Coix* was not parasitized, there was no possibility of *Cuscuta* indirectly reducing colonization of *Coix* roots by the AM fungi through competition for carbon. On the other hand, suppression of AM fungi in *Mikania* roots could have been caused by the direct effect of fungicide and indirectly through competition from *Cuscuta* for carbon. However, whether the bactericide contributed to the decline in AM fungal colonization of *Mikania* roots indirectly through altered host plant physiology or by acting directly on the fungi remains to be resolved.

In the soil where neither bactericide nor fungicide was applied, *Mikania* had a higher level of root colonization by AM fungi (58%) (Figure 3d) than *Coix* (38%) (Figure 3a). These results are counter to the notion that exotic plants are less likely than native plant species to associate with AM fungi (Bunn et al., 2015; Klironomos, 2003; Pringle et al., 2009). Although invasive plants may leave behind coevolved mutualists in the native range (Kowalski et al., 2015), as the density, range, and time‐since‐invasion increase, the plants may acquire novel microbial mutualists (the host‐jumping hypothesis; Shipunov, Newcombe, Raghavendra, & Anderson, 2008; Kowalski et al., 2015). For instance, *Cyperus rotundu*s that invaded the U.S. Gulf coast region harbored a fungal mutualist *Balansia cyperi* that was native to the region (Stovall & Clay, 1988). The fungus likely jumped from a native *Cyperus* host to *C. rotundus* (Kowalski et al., 2015). Invasive plants may also reunite with native‐range mutualists through cointroductions (the cointroduction hypothesis; Shipunov et al., 2008). For instance, communities of endophytic fungi were similar between invaded and native ranges of *Centaurea stoebe*, suggesting multiple cointroductions of different fungal species (Shipunov et al., 2008). *Pinus contorta* coinvaded New Zealand with its ectomycorrhizal fungal communities (Dickie et al., 2010). Several Australian ectomycorrhizal fungi were found in plantations of Australian *Eucalyptus* species in the Iberian Peninsula, further supporting the idea of cointroductions (Díez, 2005). In the Iberian Peninsula, the Australian *Acacia longifolia* harbored symbiotic nitrogen‐fixing bacteria that are native to Australia (Rodríguez‐Echeverría, 2010). Whether *Mikania* that has been present in China for close to 100 years (Holm et al., 1977) has acquired new microbial symbionts and/or reunited with those in its native range remains an area of further study.

The number of CFUs of soil bacteria was highest when *Mikania* was heavily parasitized by *Cuscuta* and the soil fungi suppressed and in the presence of a full complement of soil bacteria (Figure 4c). These findings support the idea that the impacts of parasitic plants on their hosts can trigger indirect interactions between parasitic plants and other species in the community (Pennings & Callaway, 2002). It is likely that heavy parasitism by *Cuscuta* caused an increase in *Mikania* root exudates that in turn promoted bacterial growth in the soil. Root‐derived exudates are a major source of carbon and nutrients for soil bacterial community (Dennis, Miller, & Hirsch, 2010). It is thought that parasitized hosts may increase allocation of resources into the roots, but evidence is scarce and conflicting (Quested, 2008). In a mixed grassland community, infection by a root hemiparasite *R. minor* stimulated the activity of belowground decomposers, which was attributed to enhanced supply of substrates because the host's root exudation increased (Bardgett et al., 2006). The same study reported a reduced fungal‐to‐bacterial ratio in the presence of the hemiparasite (Bardgett et al., 2006). Soil heterotrophic microbial communities tended to become more abundant and functionally even beneath *Pinus nigra* trees that were parasitized by mistletoe (*Viscum album* subsp. *austriacum*) than beneath nonparasitized trees (Mellado, Morillas, Gallardo, & Zamora, 2016). In contrast, parasitism by *C. campestris* on *Mikania* caused a decrease in soil microbial biomass and altered functional diversity of soil microbial communities underneath the invader (Li et al., 2014). Thus, by altering soil microbial biomass and diversity, parasitic plants could influence key soil functions that are driven my microbial communities (e.g., decomposition and nutrient release), which may ultimately influence the growth of native plants around parasitized invasive plants.

It is also likely that the fungicide contributed to an increase in the number of CFUs of soil bacteria (Figure 4c) by suppressing competitive effects of soil fungi on bacteria. Intermicrobial competition occurs in many natural ecosystems and may arise due to limiting nutrients and space, resulting in the reduced growth of some species, and a change in microbial community composition (Bell, Callender, Whyte, & Greer, 2013). This may feedback on plant growth because different components of the microbial community may exert differential effects on plant growth (Bever, Platt, & Morton, 2012). Competitive interactions between fungi and soil bacteria have been observed (Fitter & Garbaye, 1994; Liu, Yu, Xie, & Staehelin, 2016). For instance, suppression of pathogenic fungi (*Fusarium oxysporum*) by application of fungicides promoted activities of nitrogen‐fixing bacteria in the roots of *Ormosia glaberrima* seedlings (Liu et al., 2016). Hence, it is likely that in our case, the fungicide suppressed soil fungi, which in turn freed the soil bacteria from fungal competition.

### 4.2 Conclusion and implication of the findings for the management of *Mikania*

We found that the native holoparasitic plant *Cuscuta* and soil microbes had synergistic suppressive effects on growth of the invader *Mikania*, while the native *Coix* benefitted from such interactions. Our results suggest that *Cuscuta* may be used in combination with soil microbes to control *Mikania*. Practitioners of classical biological are often faced with the challenge of achieving a successful control of invaders at minimal environmental cost (Müller‐Schärer & Schaffner, 2008). Therefore, the native *Cuscuta* may be a viable alternative to importation of new species to control *Mikania*. However, as the soil fungi and bacteria modified the effect of *Cuscuta*, the identity and impact of the soil microbial community should be an important consideration. Thus, we suggest that future studies should identify the lineage‐specific soil‐borne pathogens and mutualists that may be useful in management of *Mikania* in combination with *Cuscusta*.

Since parasitic plants selectively depress the biomass of preferred host taxa that may be competitively dominant within a community, plant parasitism can alter the competitive balance between preferred and nonpreferred hosts (Pennings & Callaway, 2002). As a result of this indirect effect, parasitic plants can alter plant community biomass, species composition, and dynamics (Pennings & Callaway, 2002). For instance, field observations and experimental removal of *C. salina* from a Northern Californian salt marsh found that the parasite reduced the abundance of dominant host species in the community and facilitated plant species evenness, richness, and diversity (Grewell, 2008; Pennings & Callaway, 1996). A perturbation field experiment at two sites in England (Holme and Strumpshaw) found that *R. minor* structured a grassland community by selectively parasitizing components of the flora and modifying competitive interactions between plants (Gibson & Watkinson, 1992). Empirical studies have shown that the direction and magnitude of effects of parasitic plants may be influenced by environmental contexts like plant community composition, nutrient and moisture availability, and mycorrhizal fungi present (Le, Tennakoon, Metali, Lim, & Bolin, 2015; Matthies & Egli, 1999; Pennings & Callaway, 1996; Stein et al., 2009; Těšitel, Těšitelová, Fisher, Lepš, & Cameron, 2015). Because of the biotic and abiotic complexity inherent in ecological communities, the present results of a pot and mesocosm study should be corroborated by studies that are conducted under more complex ecological conditions in the field.

## CONFLICT OF INTEREST

The authors declare there is no conflict of interest.

## AUTHOR CONTRIBUTIONS

JL and MD conceived and designed the experiment; JL conducted the experiment and analyzed the data; JL, AMOO, FHY, and MD wrote, revised, and approved the manuscript.

## DATA AVAILABILITY

The data have been deposited in Dryad with https://doi.org/10.5061/dryad.92kr452.
