# Supplementary material for: A native parasitic plant and soil microorganisms facilitate a native plant co‐occurrence with an invasive plant
Source: Ecol Evol. 2019 Jul 4;9(15):8652–63. doi: 10.1002/ece3.5407 (PMC6686308; doi:10.1002/ece3.5407)

**SUPPLEMENTARY ONLINE MATERIAL**

**Tables**

| Effect | *F* | *P* |
| --- | --- | --- |
| ***Main*** |  |  |
| Parasitism (P) | **157.166** | **<0.001** |
| Bactericide (B) | 2.042 | 0.161 |
| Fungicide (F) | **16.029** | **<0.001** |
| ***Interaction*** |  |  |
| P × B | **18.778** | **<0.001** |
| P × F | **11.307** | **<0.001** |
| B × F | **8.017** | **0.008** |
| P × B × F | **24.839** | **<0.001** |

**Table S1.** Results of a three-way analysis of variance testing for the main and interactive effects of a parasitism on the invasive plant *Mikania micrantha* by *Cuscuta campestris* (P), bactericide (B), and fungicide (F) on the biomass of invasive *M. micrantha* that was grown together with a native plant *Coix lacryma-jobi*. Figures highlighted in bold print are statistically significant (α=0.05).

**Table S2.** Results of a three-way analysis of variance testing for the main and interactive effects of a parasitism on the invasive plant *Mikania micrantha* by *Cuscuta campestris* (P), bactericide (B), and fungicide (F) on the biomass of a native plant *Coix lacryma-jobi* that was grown with *M. micrantha*. Figures highlighted in bold print are statistically significant (α=0.05).

| Effect | *F* | *P* |
| --- | --- | --- |
| ***Main*** |  |  |
| Parasitism (P) | **26.594** | **<0.001** |
| Bactericide (B) | **7.012** | **0.012** |
| Fungicide (F) | **68.837** | **<0.001** |
| ***Interaction*** |  |  |
| P × B | 1.839 | 0.175 |
| P × F | **3.763** | **0.034** |
| B × F | 1.092 | 0.304 |
| P × B × F | **9.002** | **0.001** |

**Table S3.** Results of a three-way analysis of variance testing for the main and interactive effects of a parasitism on the invasive plant *Mikania micrantha* by *Cuscuta campestris* (P), bactericide (B), and fungicide (F) on the level of colonization of *M. micrantha* roots by AM fungi. Figures highlighted in bold print are statistically significant (α=0.05).

| Effect | *F* | *P* |
| --- | --- | --- |
| ***Main*** |  |  |
| Parasitism (P) | 1.073 | 0.355 |
| Bactericide (B) | 1.718 | 0.200 |
| Fungicide (F) | **91.265** | **<0.001** |
| ***Interaction*** |  |  |
| P × B | 0.289 | 0.751 |
| P × F | **4.474** | **0.020** |
| B × F | 0.473 | 0.497 |
| P × B × F | **5.461** | **0.010** |

**Table S4.** Results of a three-way analysis of variance testing for the main and interactive effects of a parasitism on the invasive plant *Mikania micrantha* by *Cuscuta campestris* (P), bactericide (B), and fungicide (F) on the level of colonization of *Coix lacryma-jobi* roots by AM fungi. Figures highlighted in bold print are statistically significant (α=0.05).

| Effect | *F* | *P* |
| --- | --- | --- |
| ***Main*** |  |  |
| Parasitism (P) | 0.638 | 0.536 |
| Bactericide (B) | 0.014 | 0.908 |
| Fungicide (F) | **48.848** | **<0.001** |
| ***Interaction*** |  |  |
| P × B | 0.910 | 0.414 |
| P × F | 2.465 | 0.103 |
| B × F | 0.990 | 0.328 |
| P × B × F | 0.639 | 0.535 |

**Table S5.** Results of a three-way analysis of variance testing for the main and interactive effects of a parasitism on the invasive plant *Mikania micrantha* by *Cuscuta campestris* (P), bactericide (B), and fungicide (F) on the number of colony forming units of soil bacteria in pots where *M. micrantha* and the native plant *Coix lacryma-jobi* were grown together. Figures highlighted in bold print are statistically significant (α=0.05).

| Effect | *F* | *P* |
| --- | --- | --- |
| ***Main*** |  |  |
| Parasitism (P) | 0.799 | 0.468 |
| Bactericide (B) | **8.365** | **0.007** |
| Fungicide (F) | 0.763 | 0.389 |
| ***Interaction*** |  |  |
| P × B | 2.068 | 0.144 |
| P × F | 0.098 | 0.907 |
| B × F | **8.022** | **0.008** |
| P × B × F | **4.737** | **0.016** |

**Figure S1.** A photograph of an experimental set in which an invasive plant *Mikania micrantha* was grown with a native plant *Coix lacryma-jobi* when *M. micrantha* was parasitized by *Cuscuta campestris*. *Mikania micrantha* is on the left and has broad leaves, while *C. lacryma-jobi* is on the right and has narrow leaves. *Cuscuta campestris* has a yellowish stem wound around *M. micrantha*.


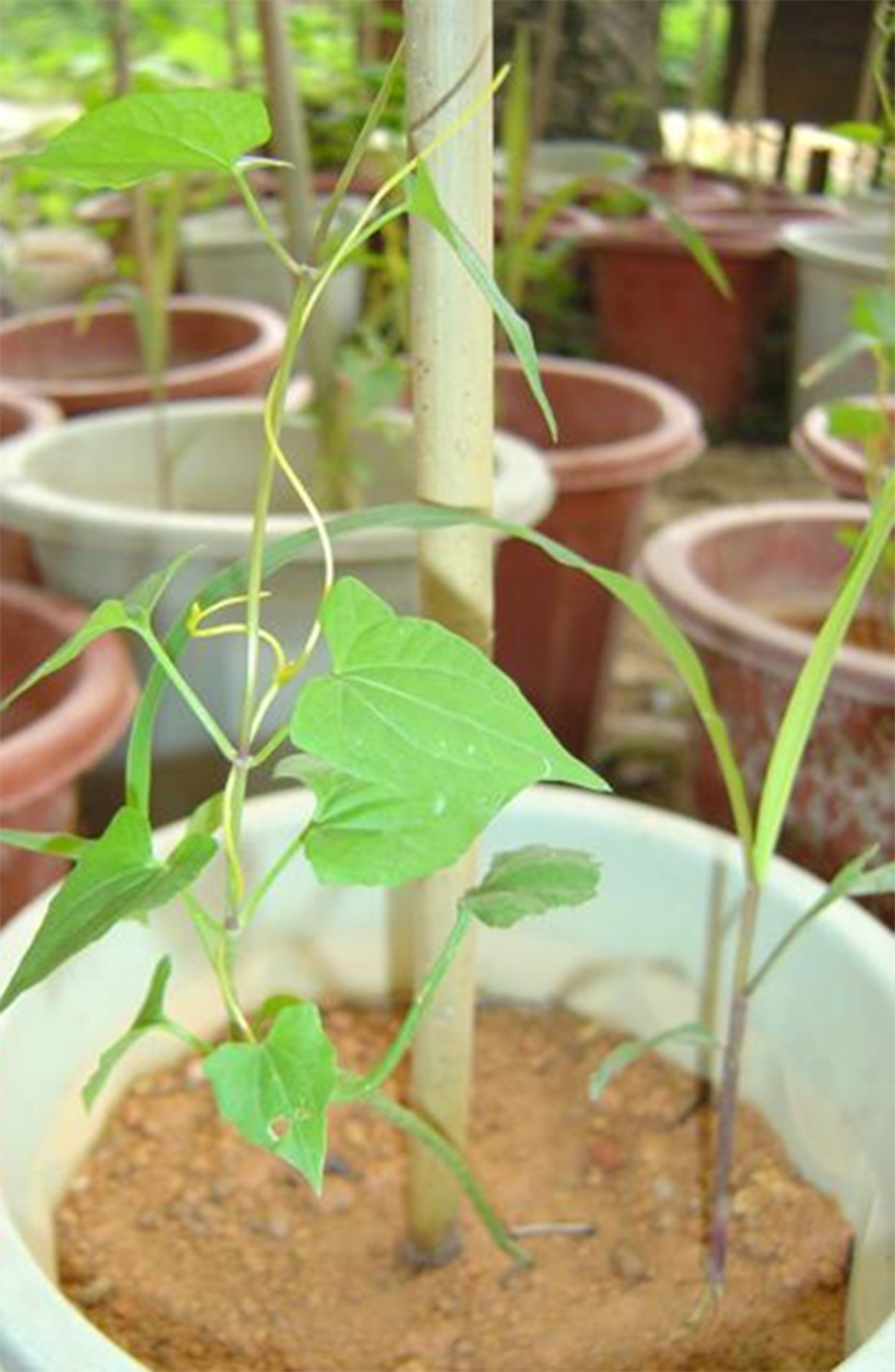

Supplement: Supplementary file 1 [file ECE3-9-8652-s001.docx]
